# *Brevundimonas* spp: Emerging global opportunistic pathogens

**DOI:** 10.1080/21505594.2017.1419116

**Published:** 2018-02-27

**Authors:** Michael P. Ryan, J. Tony Pembroke

**Affiliations:** a Industrial Biochemistry Programme, Department of Chemical Sciences, School of Natural Sciences, University of Limerick, Limerick, Ireland; b Molecular Biochemistry Laboratory, Department of Chemical Sciences, School of Natural Sciences, University of Limerick, Limerick, Ireland; c Bernal Institute, University of Limerick, Limerick, Ireland

**Keywords:** Brevundimonas, non-fermenting nosocomial infection, environmental bacteria

## Abstract

Non-fermenting Gram-negative bacteria are problematic in clinical locations, being one of the most prevalent causes of nosocomial infections. Many of these non-fermenting Gram-negative bacteria are opportunistic pathogens that affect patients that are suffering with underlying medical conditions and diseases. *Brevundimonas* spp., in particular *Brevundimonas diminuta* and *Brevundimonas vesicularis*, are a genus of non-fermenting Gram-negative bacteria considered of minor clinical importance. Forty-nine separate instances of infection relating to *Brevundimonas* spp were found in the scientific literature along with two pseudo-infections. The majority of these instances were infection with *Brevundimonas vesicularis* (thirty-five cases – 71%). The major condition associated with *Brevundimonas* spp infection was bacteraemia with seventeen individual cases/outbreaks (35%). This review identified forty-nine examples of *Brevundimonas* spp. infections have been discussed in the literature. These findings indicate that infection review programs should consider investigation of possible *Brevundimonas* spp outbreaks if these bacteria are clinically isolated in more than one patient.

## Introduction

Gram-negative, non-fermenting bacteria are an emerging concern in clinical locations, being a common cause of nosocomial infections. Opportunistic pathogens from this group include many different bacterial species, including: *Acinetobacter baumannii*, *Burkholderia cepacia*, *Ralstonia pickettii*, *Pseudomonas aeruginosa*, *Sphingomonas pauciomobilis*, and *Stenotrophomonas maltophilia* [1–8]. The group can survive in a wide variety of environments including different water sources (aircraft water, bottled water, hospital water, purified water) [9–12], and are usually resistant to a wide array of antimicrobials [13,14]. Examples include resistance to penicillins, aminoglycosides and monobactems in *R. pickettii* [13] and penicillins, aminoglycosides, carbapenems and monobactems in *S. maltophilia* [14]. Bacteria such as these have the ability to infect patients/individuals with underlying medical conditions and diseases. Examination of the scientific literature showed multiple types of infections resulting from *Brevundimonas* spp. This indicates that the genus may be a more widespread pathogen than was hitherto thought, with infections caused by *Brevundimonas* spp. being invasive and severe. The goal of this study was to give an overview of the range of *Brevundimonas* spp infections, any underlying conditions associated with *Brevundimonas* spp infections and the treatment options used in the treatment of any *Brevundimonas* spp infections in order to assist medical practitioners.

### Genus Brevundimonas

The genus *Brevundimonas* was first proposed by Segers et al [15]; incorporating *Pseudomonas diminuta* and *Pseudomonas vesicularis* [16,17]. Several species of the genus *Caulobacter* were later transferred to *Brevundimonas* significantly emending the description of the genus [18]. Currently, there are 25 species with valid published names within the *Brevundimonas* genus (http://www.bacterio.net/brevundimonas.html). The type species is *Brevundimonas diminuta*; with the type strain being LMG 2089.

*Brevundimonas* species are aerobic Gram-negative, oxidase and catalase positive, non-fermenting rods 1 to 4 μm in length and 0.5 μm in width, belonging to the Alphaproteobacteria class and Caulobacteraceae family with a DNA G + C content of 65% to 68% [15]. Motility is provided by one short polar flagellum. *Brevundimonas* spp. have been isolated from multiple environments, including soils [9–21], deep subsea floor sediment [22] activated sludge,[23] black sand, [24], deep subsea floor sediment [25] numerous aquatic habitats [26], purified water [27] and also from the condensation water of a Russian space laboratory [28].

## Brevundimonas spp.

### Brevundimonas diminuta

*Brevundimonas diminuta* is the type species of the *Brevundimonas* genus. It has been isolated from clinical specimens, including blood and urine [15] as well as from the lung sputum of cystic fibrosis patients [29]. *B. diminuta* is not believed to be a significant pathogen and its virulence is generally low. *B. diminuta* is used as a test organism to validate reverse-osmosis (RO) filtration devices for drinking water purification and is also used to test the porosity of pharmaceutical-grade filters (0.2 μm) due to the small size of the bacterium when grown in minimal media [30,31]. The bacterium has however been shown to be capable of penetrating these filters [32]. The bacterium has been used as a potential bioremediator of marine oil pollution including diesels, n-alkanes and polycyclic aromatic hydrocarbons [33,34] and insecticides [35]. *B. diminuta* has also been used to mitigate the toxic effects of heavy metals on plant growth (rice) in contaminated soils [36]. *B. diminuta* also possesses the ability to survive sanitizers such as Hydrogen Peroxide + Peracetic Acid [37]. All available reported incidences of infection credited to *B. diminuta* are listed in Table 1-3.

**Table 1.** Incidences of *Brevundimonas* spp. infection from 1978–2000 – Main characteristics of the case reports.

Author (Ref)/ Species	Year	Sex/Age	Country	Co-morbidity	Type of infection	Susceptible to	Resistance to	Antibiotic treatment	Outcome
Otto et al. [53], *B. vesicularis*	1978	Multiple cases (5 cases)	USA	N/A	Cervicitis	Ampicillin, Carbenicillin, Gentamicin, Kanamycin Nitrofurantoin, Streptomycin, Tetracycline	Colistin, Nalidixic acid, Sulfisoxazole	N/A	N/A
Vanholder et al. [54], *B. vesicularis*	1990	M/62	Belgium	Hemodialysis,	Bacteraemia/HA	N/A	N/A	Cefotaxime, Tobramycin	Complete recovery
Vanholder et al. [55], *B. vesicularis*	1992	F/62	Belgium	Hemodialysis,	Bacteraemia/HA	N/A	N/A	Cefotaxime, Tobramycin	Complete recovery
Planes et al. [56], *B. vesicularis*	1992	W/54	USA	Systemic lupus erythematosus and chronic active autoimmune hepatitis	Bacteraemia/HA	N/A	N/A	Ceftazidime, Tobramycin	Surgical resection of the infected tissue
Pasadakis et al. [57], *B. diminuta*	1993	N/A	Greece	End-stage renal failure	Peritonitis	N/A	N/A	Initial 500 mg/L ceftazidime in a I-L + 1.7 mg/kg of tobramycin. Maintenance doses 250 mg/2 L of ceftazidime + 16 mg/2 L of tobramycin	Complete recovery
Oberhelman et al. [58], *B. vesicularis*	1994	M/5	USA	Sickle cell anaemia	Pneumonia/CA	N/A	N/A	Ceftriaxone, Gentamicin	Complete recovery
Calegari et al. [59], *B. vesicularis*	1996	M/60	Uruguay	Trauma	Botryomycosis/CA	N/A	N/A	Cefuroxime	Complete recovery
Gilad et al. [60], *B. vesicularis*	2000	F/42	Israel	Mitral valve replacement	Bacteraemia/HA	Amoxicillin-Clavulanate, Amino-glycosides, Co-trimoxazole, Imipenem, Mezlocillin, Piperacillin, Piperacillin- Tazobactam	Ampicillin, Aztreonam, Cefuroxime, Ceftriaxone, Ceftazidime, Ciprofloxacin	Piperacillin- Tazobactam	Complete recovery

M- Male, F- Female, N/A – Not Available, CA – Community Acquired, HA- Hospital Acquired.

### Brevundimonas vesicularis

*Brevundimonas vesicularis* has been isolated from eye, urine, wound cultures, the central nervous system, cervical specimens [38], and also been found in the lung sputum of cystic fibrosis patients [39]. The organism has been shown to support the growth of *Legionella* in nutrient limited water conditions [40]. The mechanism behind this phenomenon has not been elucidated but it is hypothesised to be due to cryptic growth, with *B. vesicularis* having the ability to grow in nutrient limited conditions and *Legionella* growing on this [40]. Further research is required to gain a fuller understanding of this phenomenon. *B. vesicularis* has been used as a potential bioremediator of polyaromatic hydrocarbons [41]. All reported incidences of infection credited to *B. vesicularis* are listed in Table 1–3.

### Identification of* Brevundimonas* spp

Members of the *Brevundimonas* spp. are Gram negative with cells appearing as straight slim rods upon Gram staining. They are non-spore forming. They are aerobic with optimal growth temperatures of between 30–37°C. They are oxidase positive and give variable results for catalase (usually positive). *B. diminuta* colonies have a chalk white appearance on MacConkey agar, whereas *B. vesicularis* colonies have an orange colour given by an intracellular pigment. Both grow slowly on ordinary nutrient media [42]. Both *B. vesicularis* and *B. diminuta* can be identified via commercial biochemical identification kits or systems such as the API 20 NE system, the VITEK 2 system (bioMerieux) or the Phoenix-100 automated system (Becton Dickinson). MALDI-TOF identification is also being used for identification of *Brevundimonas* spp. in clinical situations [43,44]. Species specific Real Time PCR primers and Fluorescence *in situ* hybridization (FISH) probes have been designed for *B. diminuta* [45]. These can be seen in Table 4.

## Factors associated with Infection

### Underlying causes

The majority of infections with *Brevundimonas* (Table 1–3) were found to have an underlying condition or disease that allowed patients to succumb to *Brevundimonas* infection. Seven patients, who were suffering with various types of cancer, contracted *Brevundimonas* -related bacteraemia, Urinary Tract Infection (UTI) and Empyema [46]; a 56-year-old female with *Lupus glomerulonephritis* acquired a *Brevundimonas* -related leg ulcer [47] and an infant suffering from Pompe disease was diagnosed with *Brevundimonas* -related bacteraemia [48]. Other examples of patients infected with *Brevundimonas* having underlying conditions are shown in Table 1–3. Such examples demonstrate the role of *Brevundimonas* as an opportunistic pathogen in immunocompromised individuals. Many of these instances of infection were hospital acquired although a large number were community acquired, which is interesting as opportunistic pathogens such as *Brevundimonas* spp or *R. pickettii* are usually contracted in hospital settings [7].

**Table 2.** Incidences of *Brevundimonas* spp. infection from 2001 – 2010. Main characteristics of the case reports.

Author (Ref)	Year	Sex/Age	Country	Co-morbidity	Type of infection	Susceptible to	Resistance to	Antibiotic treatment	Outcome
Lee et al. [61], Various	2000–2010	Multiple (30 cases)	Taiwan	Cancer patients	Bacteraemia	Ciprofloxacin, Colistin, Doripenem, Tigecycline	Amikacin, Piperacillin/tazobactam	Cefotaxime, Ceftazidime, Cefmetazole, Cefazolin, Cefuroxime, Ceftriaxone, Imipenem, Piperacillin/tazobactam, Ticaracillin-Clavulanate	Complete recovery
Seve et al. [62], *B. diminuta*	2004	F/35	France	Leukaemia	Bacteraemia/HA	Ciprofloxacin, Imipenem	Amikacin, Cefepime and Ceftazidime, Piperacillin	Initially Cefepime, Amikacin After susceptibility testing: Ciprofloxacin, Imipenem	Complete recovery
Chi et al. [63], *B. vesicularis*	2004	M/38	Taiwan	None	Tonsillitis/CA	Cefoperazone.	Ampicillin, Aztreonam, Cefazolin, Ceftazidime, Ciprofloxacin, Flomoxef, Gentamicin, Tobramycin	Amoxicillin/ Clavulanic acid	Complete recovery
Chi et al. [63], *B. diminuta*	2004	M/62	Taiwan	Liver cirrhosis, Encephalopathy, Spontaneous bacterial peritonitis	Bloodstream infection/CA	Amikacin, Aztreonam, Cefotaxime, Cefepime, Chloramphenicol, Ciprofloxacin Flomoxef, Gentamicin, Imipenem, Piperacillin-Tazobactam, Tetracycline, Tobramycin, Co-trimoxazole	Ampicillin, Cefazolin, Cefoperazone, Ceftazidime, Ceftriaxone	Cefotaxime	Complete recovery
Han et al., [46], *B. diminuta*	2005	Multiple (7 Cases)	USA	Cancer	Bacteraemia, Urinary Tract Infection, Empyema/HA	Amikacin, Imipenem and Ticarcillin/clavulanate	Ampicillin, Cefepime, Ciprofloxacin	Cefepime, Imipenem, Levofloxacin, Meropenem, Nafcillin, Tobramycin, Ticarcillin/ clavulanate, Vancomycin	Complete recovery
Karadag et al. [38], *B. vesicularis*	2005–2011	Multiple (8 cases)	Turkey	Neonates	Septicaemia/HA	Amikacin, Imipenem,	Aztreonam, Ceftazidime, Piperacillin/tazobactam	Ampicillin, Cefotaxime, Ciprofloxacin, Meropenem,	7 Complete recovery, 1 Died
Vahid [64] *B. vesicularis*	2005	W/36	USA	Acute myelogenous leukaemia	Bacteraemia	Ciprofloxacin, Ticaracillin-Clavulanate	Amikacin, Aztreonam, Cefepime, Ceftazidime, Ceftriaxone, Meropenem, Piperacillin/tazobactam	Initially: Amikacin, Ampohotericin B, Gancyclovir Vancomycin	Died
Papaefstathiou et al., [65], *B. vesicularis*	2005	F/92	Greece	Cardiac failure	Bacteraemia/CA	Amoxicillin-clavulanate, Aminoglycosides, Azlocillin, Aztreonam Second and Third-generation Cephalosporins, Imipenem, Piperacillin, Tetracycline, Trimethoprim-Sulfamethoxazole	Ampicillin, Cephalothin, Ciprofloxacin	Cefuroxime, Netilmicin	Died
Niedermeier et al. [66], *B. vesicularis*	2005	F/37	USA	Acute myeloid leukemia, Pregnancy, Pancytopenia	Sepsis/HA	N/A	N/A	Clindamycin, Piperacillin-tazobactam	Complete recovery from sepsis
Mondello et al. [67], *B. vesicularis*	2006	M/24	Italy	Pilocytic astrocytoma	Meningitis/HA	Ciprofloxacin, Co-trimoxazole, Tetracycline	N/A	Initially: Ceftriaxone, Ciprofloxacin, Co-trimoxazole After Treatment failure: Amikacin	Complete recovery
Choi et al. [68], *B. vesicularis*	2006	M/55	South Korea	Diabetes, Continuous ambulatory peritoneal dialysis	Peritonitis/CA	N/A	N/A	Aztreonam, Cefazolin, Ceftazidime, Ciprofloxacin, Vancomycin	Complete recovery
Yang et al. [69], *B. vesicularis*	2006	M/40	Taiwan	None	Endocarditis/ CA	Amikacin, Amoxicillin, Gentamicin, Piperacillin, Aztreonam, Cefepime, Meropenem, Netilmicin, Ampicillin, Ciprofloxacin, Cefazolin, Cefmetazole, Ceftazidime, Cefotaxime, Ceftriaxone, Ticarcillin	N/A	Cefazolin, Gentamicin	Complete recovery
Zhang et al. [70], *B. vesicularis*	2006–2009	Multiple Cases (22 patients)	Taiwan	Various (Cancer, heart failure, COPD, Kidney diease)	Bacteraemia/CA/HA	Amikacin, Cefpirome, Imipenem, Piperacillin/tazobactam,	N/A	Various (Penicillin's, Cephalosporins)	Complete recovery in 21 cases. 1 case of death
Pelletier et al. [71], *B. vesicularis*	2007	F/45	USA	None	Keratitis	Ceftazidime, Ciprofloxacin, Gentamicin, Levofloxacin	N/A	Ceftazidime	Complete recovery
Sofer et al., [72], *B. vesicularis*	2007	F/15 Month old	Israel	None	Septic Arthritis/CA	Aminoglycosides, Aminopenicillins, Cephalposporins, Piperacillin, Quinolones, Trimethoprim-sulfamethoxazole	N/A	Cefuroxime	Complete recovery
Menuet et al. [29], *B. vesicularis*	2008	F/17	France	Cystic Fibrosis	Pneumonia	Amikacin, Ceftriaxone, Gentamicin, Imipenem, Isepamicin, Rifampicin, Piperacillin/tazobactam, Ticarcillin, Ticarcillin- Clavulanate, Tobramycin	Amoxicillin, Amoxicillin-Clavulanate, Ceftazidime, Ciprofloxacin, Colistin, Trimethoprim-Sulfamethoxazole	Imipenem, Tobramycin	Complete recovery
Panasiti et al. [73], *B. vesicularis*	2008	M/71	Italy	None	Cutaneous Infection/CA	Amikacin, Cefoxitin, Ceftazidime, Cefuroxime, Cefalotin, Cepodoxime, Gentamycin, Tobramycin	Amoxicillin-Clavulanate, Cefeoime, Ofloxacin, Norfloxacin	Amoxicillin-Clavulanate	Complete recovery
Viswanathan et al. [74], *B. vesicularis*	2009	M/Infant	India	Newborn	Sepsis/HA	Amikacin, Cefotaxime, Ciprofloxacin, Gentamicin, Meropenem, Ofloxacin, Piperacillin tazobactam	N/A	Amikacin, Cefotaxime	Complete recovery
Chandra et al. [75], *B. vesicularis*	2010	M/31	USA	Biliary Pancreatitis	Bacteraemia	Amikacin, Cefuroxime, Cefepime, Ciprofloxacin, Gentamicin, Piperacillin/tazobactam, Polymyxin B,	Aztreonam.	Cefuroxime	Complete recovery
Restrepo et al. [76], *B. vesicularis*	2010	F/44	Columbia	None known	Reactive Arthritis + Bacteraemia	Amikacin, Imipenem, Meropenem, Piperacillin/tazobactam	Aztreonam, Ciprofloxacin	Initially: Amikacin, Ciprofloxacin Following Sensitivity testing: Piperacillin/tazobactam	Complete recovery
Estrela and Abraham [77] *B. vancanneytii*	2010	N/A	Germany	N/A	Endocarditis	N/A	N/A	N/A	N/A

M- Male, F- Female, N/A – Not Available, CA – Community Acquired, HA- Hospital Acquired.

**Table 3.** Incidences of *Brevundimonas* spp. infection from 2010 – 2017. Main characteristics of the case reports.

Author (Ref)	Year	Sex/Age	Country	Co-morbidity	Type of infection	Susceptible to	TResistance to	Antibiotic treatment	Outcome
Shang et al. [78], *B. vesicularis*	2011	M/83	Taiwan	Type 2 diabetes, Hypertension	Progressive leucocytosis/HA	Amikacin, Ampicillin/Sulbactam, Cefazolin, Ceftazidime, Ceftriaxone, Cefepime, Ertapenem, Gentamicin, Imipenem, Piperacillin/Tazobactam	Ampicillin, Ciprofloxacin	N/A	Complete recovery
Shang et al. [78], *B. vesicularis*	2011	M/25	Taiwan	Lymphoma	Febrile neutropenia/HA	Amikacin, Ampicillin/Sulbactam, Cefazolin, Ceftriaxone, Ertapenem, Gentamicin, Imipenem, Piperacillin/Tazobactam	Ampicillin, Ceftazidime, Cefepime, Ciprofloxacin	Ceftriaxone	Complete recovery
Bhatawadekar & Sharma [79] *B. vesicularis*	2011	F/Infant	India	Infant	Bacteraemia/CA	Amikacin, Amoxicillin, Cefotaxime, Ciprofloxacin, Gentamicin, First Generation Cephalosporins, Imipenem, Meropenem, Piperacillin, Ticarcillin	Ceftazidime, Cefoxitin, Co-trimaxazole,, Netilmicin	Cefotaxime	Complete recovery
Yoo et al. [80], *B. vesicularis*	2012	M/30	South Korea	N/A	Liver Abscess	Amikacin, Ampicillin-Sulbactam, Imipenem	Aztreonam, Ceftazidime, Cefepime, Ciprofloxacin	Ceftriaxone, Ampicillin-Sulbactam	Complete recovery
Almuzara et al. [47], *B. diminuta*	2012	F/56	Argentina	Lupus glomerulonephritis	Leg ulcer	Minocycline, Tigecycline	Ampicillin, Ampicillin/Sulbactam, Aztreonam, Cefalotin, Cefoxitin, Cefotaxime, Ceftazidime, Cefepime, Ciprofloxacin, Colistin, Gentamicin, Imipenem, Meropenem, Piperacillin/Tazobactam, Trimethoprim- sulfamethoxazole	Tigecycline plus imipenem.	Complete recovery
Karadag et al. [81], *B. vesicularis*	2012	M/Infant	Turkey	Neonate	Neonatal sepsis	Amikacin, Ceftriaxone, Gentamicin, Imipenem	Ampicillin, third-generation Cephalosporins, piperacillin-tazobactam	Empirical Ampicillin, Gentamicin. After susceptibility testing Meropenem followed by ciprofloxacin	Complete recovery
Khalifa et al. [48], *B. vesicularis*	2012	F/Infant	Tunisia	Pompe disease	Bacteraemia	Amikacin, Aztreonam Cefotaxime, Ceftazidime, Ciprofloxacin, Gentamicin, Imipenem, Ofloxacin, Piperacillin/ tazobactam	Ppiperacillin, Ticarcillin	Ceftazidime 100 mg / kg daily for 10 days and Amikacin 15 mg / kg daily	Complete recovery
Pandit et al. [82], *B. diminuta*	2012	F/66	USA	N/A	Keratitis/CA	Amikacin, Gentamicin, Tobramycin	Ampicillin, Cefotaxime, Ceftazidime, Ciprofloxacin, Moxifloxacin	Besifloxacin and Tobramycin, Following identification Tobramycin was changed to Gentamicin	Complete recovery
Lu et al. [45], *B. diminuta*	2013	M/38	China	None	Pleuritis	Amikacin, Chloramphenicol, Gentamicin, Cefoperazone-Sulbactam, Meropenem, Piperacillin/tazobactam, Tetracycline	Aztreonam, Ceftazidime, Cefepime, Ciprofloxacin, Levofloxacin, Trimethoprim-sulfamethoxazole	Initially: Ciprofloxacin After Treatment failure: Piperacillin/tazobactam	Complete recovery
Nandy et al. [83], *B. vesicularis*	2013	F/Infant	India	Infant	Bacteraemia	Meropenem, Ceftazidime/ Clavulanic acid, Netilimycin, Cefepime, Ampicillin/Sulbactam, Piperacillin/Tazobactam, Levofloxacin, Ciprofloxacin, Ceftazidime, Tobramycin, Gentamicin	Cotrimaxazole, Nalidixic acid	Piperacillin/ Tazobactam, Amikacin, Gentamycin, Fluconazole, Ciprofloxacin, Meropenem	Complete recovery
Shobha et al. [84], *B. diminuta*	2013	Infant	India	None	Urinary Tract Infection	Amikacin, Amoxicillin-Clavulanic acid, Cefotaxime, Cefepime, Imipenem, Ticarcillin/clavunalic acid, Trimethoprim–sulfamethoxazole	Ampicillin, Ciprofloxacin	Ticarcillin/clavulanic acid	Complete recovery
Gupta et al. [49], *B. vesicularis*	2014	M/24	India	None	Urinary Tract Infection	Minocycline, Piperacillin/tazobactam Trimethoprim–sulfamethoxazole	Amikacin, Amoxicillin, Amoxicillin-Clavulanate, Aztreonam Ceftazidime, Cefoperazone, Cefoperazone-Sulbactam, Cefoxitin, Cefotaxime, Colistin, Ertapenem, Gentamicin, Imipenem, Levofloxacin, Meropenem, Netilmicin, Norofloxacin, Tobramycin	Amikacin, Piperacillin/tazobactam	Complete recovery
Shujat et al. [85], *B. vesicularis*	2014	F	Pakistan	Gall Bladder issues	Bacteraemia	N/A	N/A	Meropenem	Complete recovery
Kishore [86] *B. vesicularis*	2014	M/51	India	Diabetes Mellitus (Type 2), Coronary Artery Disease	Bacteraemia	N/A	Ampicillin-Sulbactam	Amikacin, Amoxyclav	Complete recovery
Mahapatra et al. [87], *B. diminuta*	2014	M/35	India	N/A	Post traumatic abscess	N/A	N/A	N/A	Complete recovery
Ra et al. [88], *B. vesicularis*	2015	F/71	South Korea	End stage renal disease, Hypertension and diabetes mellitus	peritoneal dialysis-associated peritonitis	Cefepime, Cefotaxime, Gentamicin, Imipenem, Piperacillin	N/A	1-g dose of Cefazolin and 1-g dose of Ceftazidime per day	Complete recovery following catheter removal
Cao et al. [89], *B. diminuta*	2015	M/62	China	Myelodysplastic syndrome, Diabetes Mellitus (Type 2)	Bacteraemia	Ampicillin, Amikacin, Ceftriaxone, Cefepime, Cefazolin, Ceftazidime, Ciprofloxacin, Gentamicin, Imipenem, Levofloxacin, Piperacillin/tazobactam Trimethoprim–sulfamethoxazole	Aztreonam, Tobramycin	N/A	Complete recovery
Singh and Bhatia [90] *B. vesicularis*	2015	8 month old	India	Infant	Septicaemia/ CA	Amikacin, Cefoperazone, Levofloxacin, Piperacillin/tazobactam	Amoxicillin-Clavulanate, Ceftazidime	Initially: Amikacin, Ceftriaxone, Vancomycin Following Sensitivity testing: Cefoperazone, Levofloxacin,	Complete recovery
Chandra et al. [91], *B. vesicularis*	2017	M/18	India	Nephrotic syndrome	Nephrotic syndrome	imipenem, meropenem, amikacin, gentamicin, fluoroquinolones, minocycline, tigecycline, cefoperazone-sulbactam, ceftazidime, cefepime, and cotrimoxazole	Colistin	cefoperazone-sulbactam 1.5 g iv BD for 2 weeks	Complete recovery
Swain and Rout [92] *B. diminuta*	2017	M/56	India	Type-2 diabetes mellitus, hypertension with epileptic disorder	Bacteraemia	Amikacin, Ceftazidime, Ceftazidime/clavulanic acid, Cefuroxime, Ceftriaxone, Ciprofloxacin, Levofloxacin, Netilimycin	Amoxicillin/clavulanic acid	Amikacin, Ceftazidime	Complete recovery

M- Male, F- Female, N/A – Not Available, CA – Community Acquired, HA- Hospital Acquired.

**Table 4.** Molecular methods applied to identify *Brevundimonas* spp. [45].

Method	Target	Sequence	Species
Real Time PCR	gyrB	Forward Primer	*Brevundimonas diminuta*
		ATCGAGATCATGCTGCACTATGAGGG	
		Reverse Primer	
		TGTTGTTGGTGAAGCACAGCATGG	
		Real-Time Probe	
		ACGTCATCGTCATTCGCGGCCAGAA	
Real Time PCR	*rpoD*	Forward Primer AGTTCCTCAAGGCCTATTTCGGCT	*Brevundimonas diminuta*
		Reverse Primer	
		GGCTTCATTCTCGCTGAACTTGGT	
		Real-Time Probe	
		AGCGCATCAAGGAGATGGGCGT	
FISH	gyrB	AAGAAGCACAGCGTCCGCTTCGAGC	*Brevundimonas diminuta*
FISH	*rpoD*	TCAAGGCCTATTTCGGCTCGGAGAT	*Brevundimonas diminuta*

### Co-Infection

Reports of cases of co-infection with *Brevundimonas* spp and other bacteria were rare with only two instances (one individual case and four cases as part of an outbreak) of co-infection being described in the literature. Han et al described seven cases of infection with *B. diminuta* within the same outbreak, four of these cases had other microorganism's co-isolated (coagulase-negative *Staphylococcus* – bacteraemia, *Moraxella osloensis* – catheter, *Enterococcus* sp. – UTI and *Staphylococcus aureus* – empyema) [46]. Gupta et al. found co-infection (in a UTI) of *B. vesicularis* along with *Candida tropiclis* and *Acinetobacter* spp. [49].

### Pseudo-outbreaks

As can be seen in Table 5 to date only two pseudo-outbreaks have been reported with *Brevundimonas* spp. Pseudo-outbreaks can be problematic as they can result in superfluous treatments given to patients (e.g. unnecessary antibiotics or the removal of indwelling devices such as catheters) and can waste valuable time and resources in the clinical setting. The causes of pseudo-outbreaks may be due to a number of different factors such as contaminated water used in the bacterial testing procedures or contamination of materials used in laboratory testing. Kim et al [50] described how *B. diminuta* was the cause of a pseudo-outbreak in a general hospital ward in South Korea. Patients did not display symptoms associated with bacterial infection, even though the organism was detected. The source of the *B. diminuta* contamination was not discovered. Lee *et al.* [51]. described *B. diminuta* as the cause of pseudo-outbreak in a tertiary care centre in the USA. The contamination was traced to pre-prepared inoculant media (used in the testing procedures for bacterial detection).

**Table 5.** Incidences of *Brevundimonas* spp. Pseudo-infection from 1978 – 2017. Main characteristics of the case reports.

Author (Ref)	Year	Sex/Age	Country	Co-morbidity	Type of infection	Susceptible to	Resistance to	Antibiotic treatment	Outcome
Kim et al. [50], *B. diminuta*	2011	Multiple (3 cases)	South Korea	Multiple	Pseudobacteraemia	N/A	Amikacin, Ciprofloxacin, Colistin, Ceftazidime, Cefepime, Cefotaxime Imipenem, Piperacillin / Tazobactam, Tobramycin	Ampicillin / sulbactam, Cefpiran, Metronidazole, Netilmicin	N/A
Lee et al. [51], *B. diminuta*	2017	Multiple (12 cases)	USA	Multiple	Pesudo-infection	Levofloxain, Meropenem, Piperacillin/tazobactam, Trimethoprim–sulfamethoxazole	Ceftazidime	N/A	N/A

M- Male, F- Female, N/A – Not Available

### Treatment

The treatment of *Brevundimonas* spp. infections is frequently difficult, as these bacteria can be resistant to many different antibiotics including β-lactams and fluoroquinolones [46,47]. There have been no controlled trials of antimicrobial therapy for *Brevundimonas* spp. infections in humans therefore therapy should be informed by the results of *in vitro* susceptibility testing on isolates. In the majority of cases listed in Table 1–3 cephalosporins, penicillins or aminoglycoside antibiotics were given to treat patients and these were mostly successful.

Little is known about resistance mechanisms in *Brevundimonas* spp. Resistance to the fluoroquinolone family of antibiotics has been detected in outbreaks due to mutations in the quinolone resistance-determining region (QRDR) of the host *gyrA*, gyrB and *parC* genes [46]. Bla_VIM-2_ and Bla_VIM-13_, which mediate resistance to almost all β-lactams (except aztreonam), have been found in both environmental and clinical isolates of *B. diminuta* [47]. The presence of *Bla*_VIM-2_ is related to a Tn*1721-*class 1 integron which was discovered in all *B. diminuta* isolates, with the determinant located on a plasmid [47]. This integron also had an *aac* (*6*′)-*Ib* gene, which mediates resistance to aminoglycoside antibiotics. Tetracycline resistance genes have also been found in environmental isolates of *B. diminuta* [52].

### Breakdown of cases of infection with Brevundimonas spp.

Literature searches presented in Table 1–3 illustrate 49 separate instances of infection relating to *Brevundimonas* spp. The majority of these instances were infection with *B. vesicularis* (thirty-five cases – 71%). One outbreak had both *B. vesicularis* and *B. diminuta* and one case of infection with *B. vancanneytii* was reported. The rest of the cases were made up *B. diminuta* infections (twelve cases- 24%). The major breakdown of condition were as follows: seventeen instances of bacteraemia (34%), five instances of septicaemia/sepsis (10%), three instances of pneumonia/ pleuritis (6%), two instances each endocarditis (4%), keratitis (4%), and urinary tract infection (4%). Serious infections with *Brevundimonas* spp include four instances of septicaemia (8%), two of endocarditis (4%), one of septic arthritis (2%) and one of meningitis (2%). Other conditions include instances of two cases of tonsillitis (2%), two of liver abscess (2%) and two of botryomycosis (2%). There have also been two reported instances (4%) of *Brevundimonas* spp infection that have caused two or more conditions: bacteraemia and reactive arthritis and bacteraemia, urinary tract infection and empyema. Four instances of death have been related to *Brevundimonas* spp infection, three of bacteraemia and one of septicaemia.

### Conclusions

*Brevundimonas* spp. are not currently considered as major pathogens. However, this should be re-evaluated in light of our investigations where forty-nine examples of *Brevundimonas* spp. infections have been found in the literature. These species have characteristics, such as ability to pass through sterilising filters, which may allow them to cause potentially harmful infections and even death on occasion. Although it is of low virulence and not as big a risk as other non-fermenting Gram-negative bacteria such as *Burkholderia* etc., it should not be over looked as a possible cause of nosocomial infections and should be considered for inclusion in hospital screening and prevention programs. These programs should consider investigation of possible *Brevundimonas* spp outbreaks if these bacteria are clinically isolated in more than one patient.
